# Supplementary material for: The Alligator and the Mosquito: North American Crocodilians as Amplifiers of West Nile Virus in Changing Climates
Source: Microorganisms. 2024 Sep 14;12(9):1898. doi: 10.3390/microorganisms12091898 (PMC11433929; doi:10.3390/microorganisms12091898)
Supplement: Supplementary file 1 [file microorganisms-12-01898-s001.zip › TablesS1-S9.pdf]

Table S1: Variables used for modeling crocodilian and mosquito species in North America.

Variable	Explanation	Source
bio1	Mean diurnal range (mean of monthly temperature range)	Fick & Hijmans, 2017
bio2	Isothermality (Bio2)/(Bio7) * 100	Fick & Hijmans, 2017
bio3	Temperature seasonality (standard deviation * 100)	Fick & Hijmans, 2017
bio4	Maximum temperature of warmest month	Fick & Hijmans, 2017
bio5	Minimum temperature of coldest month	Fick & Hijmans, 2017
bio6	Temperature annual range (Bio5-Bio6)	Fick & Hijmans, 2017
bio7	Mean temperature of wettest quarter	Fick & Hijmans, 2017
bio8	Mean temperature of driest quarter	Fick & Hijmans, 2017
bio9	Mean temperature of warmest quarter	Fick & Hijmans, 2017
bio10	Mean temperature of coldest quarter	Fick & Hijmans, 2017
bio11	Annual precipitation	Fick & Hijmans, 2017
bio12	Precipitation of wettest month	Fick & Hijmans, 2017
bio13	Precipitation of driest month	Fick & Hijmans, 2017
bio14	Precipitation seasonality (coefficient of variation)	Fick & Hijmans, 2017
bio15	Precipitation of wettest quarter	Fick & Hijmans, 2017
bio16	Precipitation of driest quarter	Fick & Hijmans, 2017
bio17	Precipitation of warmest quarter	Fick & Hijmans, 2017
bio18	Precipitation of coldest quarter	Fick & Hijmans, 2017
bio19	Mean diurnal range (mean of monthly temperature range)	Fick & Hijmans, 2017
srad_01-srad_12	Monthly solar radiation	Fick & Hijmans, 2017
vapr_01-vapr_12	Monthly water vapor pressure	Fick & Hijmans, 2017
wind_01-wind_12	Monthly wind speed	Fick & Hijmans, 2017
ag_general_percent	Percent general agriculture*	MDAUS BaseVue 2013
ag_paddy_percent	Percent paddy (inundated) agriculture*	MDAUS BaseVue 2013
barren_percent	Percent barren land*	MDAUS BaseVue 2013
deciduous_percent	Percent deciduous forest*	MDAUS BaseVue 2013
evergreen_percent	Percent evergreen forest*	MDAUS BaseVue 2013
grassland_percent	Percent grassland*	MDAUS BaseVue 2013
mangrove_percent	Percent mangrove*	MDAUS BaseVue 2013
mixed_forest_percent	Percent mixed forest*	MDAUS BaseVue 2013
shrub_percent	Percent shrub or scrub*	MDAUS BaseVue 2013
urban_high_percent	Percent high density urban*	MDAUS BaseVue 2013
urban_midlow_percent	Percent low to medium density urban*	MDAUS BaseVue 2013
urban_percent	Percent combined urban*	MDAUS BaseVue 2013
water_percent	Percent water*	MDAUS BaseVue 2013
wetland_percent	Percent wetland*	MDAUS BaseVue 2013
woody_wetland_percent	Percent woody wetland*	MDAUS BaseVue 2013

*Focal statistics using 10 km radius

Table S2: *Alligator mississippiensis* model included variables with their percent contribution and permutation importance, listed in order of descending percent contribution.

Variable	Percent contribution	Permutation importance
vapr_07	39.0932	42.4386
woody_wetlands_percent	28.8367	6.9977
vapr_05	16.9782	0.5644
vapr_08	4.5319	7.5970
vapr_06	2.9862	3.1606
vapr_03	2.0561	24.4423
shrub_percent	2.0238	0.7284
vapr_01	1.6122	3.3732
bio17	0.9100	5.0886
vapr_09	0.5732	4.2650
bio4	0.2431	1.0772
wetland_percent	0.1464	0.0071
water_percent	0.0084	0.0346
srad_12	0.0006	0.2253

Table S3: *Caiman crocodilus* model included variables with their percent contribution and permutation importance, listed in order of descending percent contribution.

Variable	Percent contribution	Permutation importance
vapr_12	60.7040	1.5664
bio4	10.8655	27.3673
vapr_01	8.9976	0.0072
vapr_04	5.0080	9.2667
vapr_02	4.6505	0.0000
vapr_11	2.5979	3.0840
vapr_03	2.3527	24.6699
bio13	2.3275	6.3738
bio3	0.9626	12.1543
shrub_percent	0.3571	4.1716
wind_01	0.1931	1.9643
bio11	0.1589	1.7949
srad_03	0.1389	0.0229
wind_03	0.1297	1.8392
srad_10	0.1224	0.1957
wind_11	0.1208	1.8065
ag_general_percent	0.1060	0.2961
wind_06	0.0641	1.2797
wetland_percent	0.0623	0.5070
grassland_percent	0.0250	1.3542
water_percent	0.0133	0.0275
evergreen_percent	0.0102	0.1006
wind_10	0.0081	0.0278
wind_02	0.0068	0.0000
bio15	0.0061	0.0167
wind_07	0.0059	0.0964
wind_08	0.0028	0.0000
urban_lowmid_percent	0.0020	0.0093

Table S4: *Crocodylus acutus* model included variables with their percent contribution and permutation importance, listed in order of descending percent contribution.

Variable	Percent contribution	Permutation importance
vapr_10	49.867	48.7399
vapr_01	20.3926	0.4369
vapr_08	4.8721	0.0361
vapr_12	4.0609	19.205
vapr_02	3.9015	2.1506
bio15	3.6337	4.2844
vapr_09	3.4299	0.0368
bio4	1.7225	9.8592
water_percent	1.4859	2.8400
bio1	1.3613	0.2693
bio3	1.0475	1.2420
mangrove_percent	1.0264	0.4000
urban_high_percent	0.6925	0.9327
srad_01	0.6288	0.3975
vapr_03	0.5820	0.3057
bio16	0.2345	1.4511
grassland_percent	0.2183	1.0971
srad_11	0.1983	0.1533
srad_03	0.1862	4.5666
ag_general_percent	0.1592	0.1673
bio14	0.1046	0.9861
evergreen_percent	0.0599	0.1044
bio17	0.0590	0.1122
urban_lowmid_percent	0.0397	0.1753
shrub_percent	0.0325	0.0472
wetland_percent	0.0032	0.0032

Table S5: *Crocodylus moreletii* model included variables with their percent contribution and permutation importance, listed in order of descending percent contribution.

Variable	Percent contribution	Permutation importance
vapr_10	40.6542	40.0097
vapr_01	36.1571	5.0150
vapr_03	4.4194	19.3906
bio4	3.9428	10.2938
vapr_06	3.6267	2.4185
bio1	1.9666	3.3259
vapr_08	1.7472	0.0529
vapr_09	1.7060	0.0003
vapr_11	1.4871	0.0079
vapr_04	1.3659	10.3500
srad_04	0.6634	2.2722
bio7	0.6575	1.0008
grassland_percent	0.4388	1.3614
shrub_percent	0.3578	0.4535
woody_wetlands_percent	0.2226	1.0909
bio14	0.1697	0.7488
bio5	0.1129	0.0965
water_percent	0.0869	1.4528
wind_11	0.0719	0.2181
ag_general_percent	0.0712	0.0061
evergreen_percent	0.0364	0.0080
bio15	0.0297	0.3850
deciduous_percent	0.0041	0.0304
mangrove_percent	0.0039	0.0109

Table S6: *Culex pipiens* model included variables with their percent contribution and permutation importance, listed in order of descending percent contribution.

Variable	Percent contribution	Permutation importance
urban_percent	65.9673	29.7457
shrub_percent	16.5228	8.6996
bio11	5.9658	14.4888
vapr_03	1.7807	1.2114
vapr_02	1.4419	5.3901
srad_12	1.3916	5.6962
wind_10	1.1755	4.4475
ag_general_percent	1.1231	0.7297
srad_09	0.7403	9.0970
vapr_04	0.6698	8.0698
grassland_percent	0.4917	0.7270
evergreen_percent	0.4716	0.2015
bio7	0.4012	1.2955
vapr_07	0.3668	1.8831
bio15	0.2807	0.1208
wind_08	0.2735	1.1478
water_percent	0.2320	0.2597
wind_07	0.2223	0.3919
vapr_10	0.2189	4.8050
bio2	0.1499	0.9825
srad_03	0.0790	0.2572
bio3	0.0335	0.3521

Table S7: *Culex quinquefasciatus* model included variables with their percent contribution and permutation importance, listed in order of descending percent contribution.

Variable	Percent contribution	Permutation importance
vapr_01	46.0619	18.8598
bio4	27.0216	4.8588
bio11	13.7374	47.7966
urban_percent	3.4117	3.5353
bio6	2.0320	4.6231
ag_general_percent	1.9786	1.9858
bio3	1.3167	0.0442
vapr_03	1.0245	11.2865
mixed_forest_percent	0.6667	0.6075
urban_high_percent	0.5859	0.3681
bio2	0.5186	0.5919
vapr_08	0.4843	0.3286
bio12	0.4371	0.7819
bio10	0.3467	0.1178
vapr_04	0.2061	3.8655
deciduous_percent	0.0788	0.2670
wind_09	0.0620	0.0054
srad_04	0.0294	0.0763

Table S8: *Culex tarsalis* model included variables with their percent contribution and permutation importance, listed in order of descending percent contribution.

Variable	Percent contribution	Permutation importance
urban_percent	38.5529	17.5883
srad_09	15.7627	28.8682
shrub_percent	10.8924	3.8392
bio6	8.2565	13.5554
bio15	5.5217	2.0700
bio14	4.4283	8.0391
urban_high_percent	3.7760	1.0349
ag_general_percent	3.7377	2.0204
grassland_percent	3.5837	2.5892
deciduous_percent	1.6587	0.2204
srad_05	1.4332	2.0214
vapr_08	0.8533	2.5733
evergreen_percent	0.3990	0.0371
vapr_04	0.3891	8.9308
srad_12	0.3141	2.4509
water_percent	0.2171	0.3542
vapr_11	0.1979	3.7944
wind_05	0.0257	0.0126

Table S9: List of crocodilian farms in North America, identified through extensive web search.

Name	Purpose	Country	State	Longitude	Latitude
Crocodile Creek	Entertainment/tourism	USA	TX	-96.7518	33.47043
Gator County Adventure Park	Entertainment/tourism	USA	TX	-94.2744	29.92942
Cougar Run Ranch	Hide/meat/hunt	USA	TX	-95.1772	30.16258
Gulf Coast Gator Ranch	Entertainment/tourism	USA	MS	-89.4845	30.30496
Instagator	Entertainment/tourism	USA	LA	-90.008	30.51857
Kleibert and Sons Gator Tours	Entertainment/tourism	USA	LA	-90.4696	30.44982
Lousiana Gator Country Alligator Park	Entertainment/tourism	USA	LA	-93.1669	31.83366
Gator Chateau	Entertainment/tourism	USA	LA	-92.6667	30.24737
Gatora and Friends Alligator Park and Exotic Zoo	Entertainment/tourism	USA	LA	-94.0371	32.46558
Greenwood Gator Farm Tours	Entertainment/tourism	USA	LA	-90.9031	29.61391
Wall's Alligator Farm	Hide/meat/hunt	USA	LA	-90.6276	30.39269
C & M Gator Farm	Hide/meat/hunt	USA	LA	-90.5835	30.4379
All American Gator Products	Hide/meat/hunt	USA	FL	-80.145	26.05418
Alligator Bob's Gourmet Alligator	Hide/meat/hunt	USA	FL	-82.2963	28.07059
Alligator Inc	Hide/meat/hunt	USA	FL	-80.9179	26.75179
Brook's Brothers Alligator Farm	Entertainment/tourism	USA	FL	-81.7193	28.54722
Central Florida Trophy Hunts	Hide/meat/hunt	USA	FL	-80.7975	28.38429
Cypress Creek Farms	Hide/meat/hunt	USA	FL	-82.1791	29.96566
Everglades Gator Farm	Entertainment/tourism	USA	FL	-80.5011	25.39315
Everglades Holiday Park	Entertainment/tourism	USA	FL	-80.4445	26.06045
Gator Beach	Entertainment/tourism	USA	FL	-86.4446	30.38779
Gatorama	Entertainment/tourism	USA	FL	-81.2908	26.91857
Gatorland Inc.	Entertainment/tourism	USA	FL	-81.4024	28.3555
Jungle Adventures, Inc.	Entertainment/tourism	USA	FL	-80.977	28.53924
Okeechobee Outfitters	Hide/meat/hunt	USA	FL	-81.2121	27.50382
Parker Island Gator Farm	Hide/meat/hunt	USA	FL	-81.2841	27.22477
St. Augustine Alligator Farm Zoological Park	Entertainment/tourism	USA	FL	-81.2886	29.88181
Townsend & Sons	Hide/meat/hunt	USA	FL	-81.5611	26.79571
Vaughn's Gators	Hide/meat/hunt	USA	FL	-84.0108	30.53378
Wild Florida	Entertainment/tourism	USA	FL	-81.3031	28.08403
Croc Encounters	Entertainment/tourism	USA	FL	-82.3473	28.02671
Arkansas Alligator Farm and Petting Zoo	Entertainment/tourism	USA	AR	-93.0725	34.5152
Alligator Alley	Hide/meat/hunt	USA	AL	-87.7002	30.51751
Donald Farms	Hide/meat/hunt	USA	LA	-92.24	32.54
Lone Star Alligator Farms	Hide/meat/hunt	USA	TX	-94.4	29.83
Glass Enterprises, Inc	Hide/meat/hunt	USA	GA	-84.2	31.23
WG Alligator Farm	Hide/meat/hunt	USA	LA	-90.63	30.41
Savoie's Alligator Farm	Both (meat/tourism)	USA	LA	-90.34	29.47
Just Gators	Hide/meat/hunt	USA	LA	-90.82	29.59
Deep South Gators	Hide/meat/hunt	USA	LA	-92.26	29.98
Golden Ranch Farms	Hide/meat/hunt	USA	LA	-90.46	29.68
South Coast Alligator	Hide/meat/hunt	USA	LA	-93.69	30.34
Waterloo Ranch Ventures	Hide/meat/hunt	USA	TX	-94.39	29.81
Audacit	Hide/meat/hunt	USA	TX	-94.4	29.83
The El Cora Crocodile Sanctuary	Entertainment/tourism	Mexico		-105.3	20.73
Cocodrilario La Manzanilla	Entertainment/tourism	Mexico		-104.79	19.29
Crocodile Farm's Hummingbird Antigua	Both (meat/tourism)	Mexico		-96.34	19.29
Croco Cun Zoo	Entertainment/tourism	Mexico		-86.87	20.88
Cocodrilario Kiekari	Entertainment/tourism	Mexico		-105.22	21.52
Granja De Cocodrilos Itzamkanac	Entertainment/tourism	Mexico		-88.16	21.44
Centro de Estudios Tecnolo´- gicos del Mar No 2	Other	Mexico		-90.56	19.82
Empresa COCODRILOS CLAL CONTINENTAL	Other	Honduras		-87.95	15.32
