# Supplementary figures and images for: The Alligator and the Mosquito: North American Crocodilians as Amplifiers of West Nile Virus in Changing Climates
Source: Microorganisms. 2024 Sep 14;12(9):1898. doi: 10.3390/microorganisms12091898 (PMC11433929; doi:10.3390/microorganisms12091898)

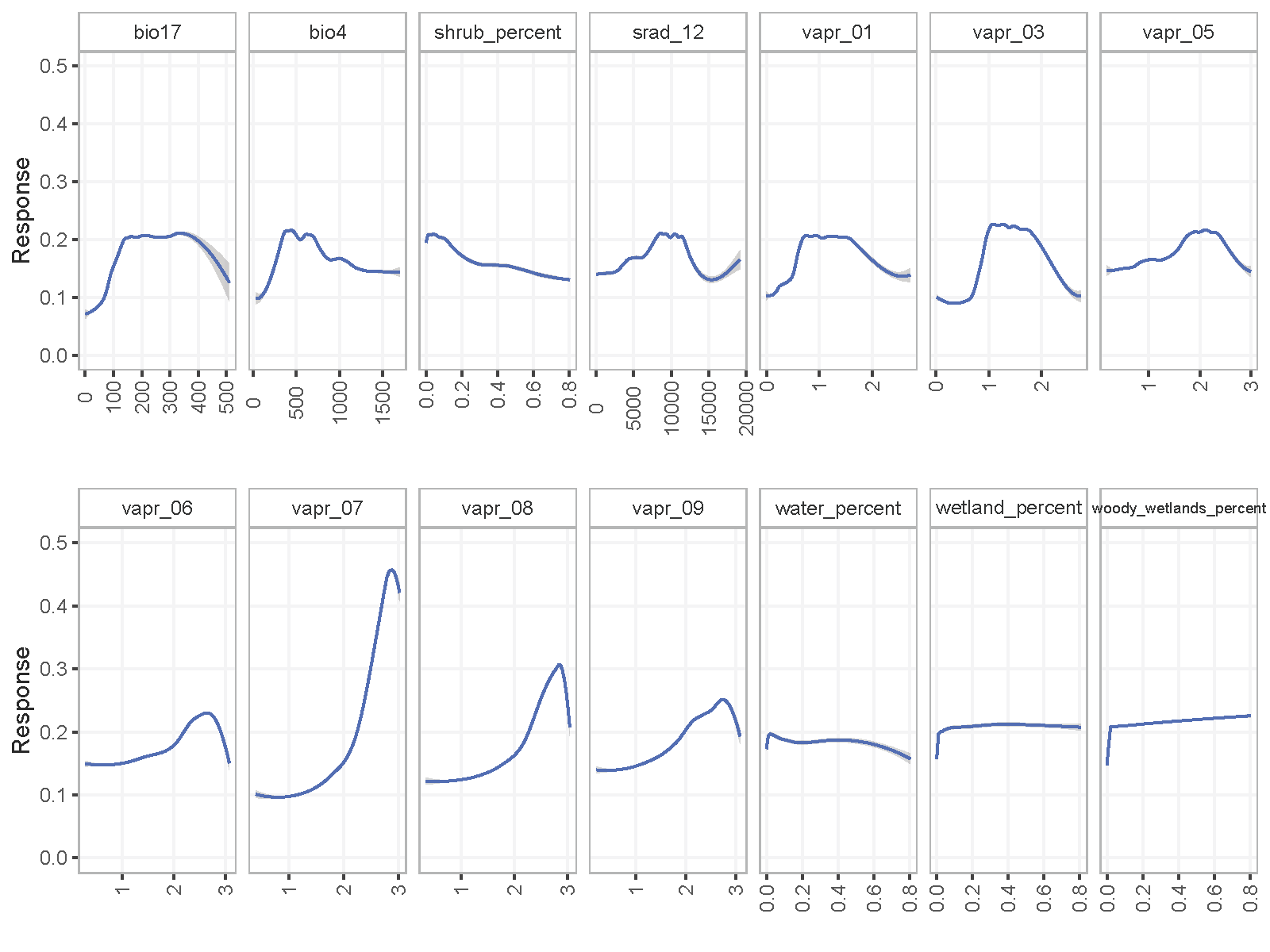

Supplement: Supplementary file 1 [file microorganisms-12-01898-s001.zip › FigureS1.png]

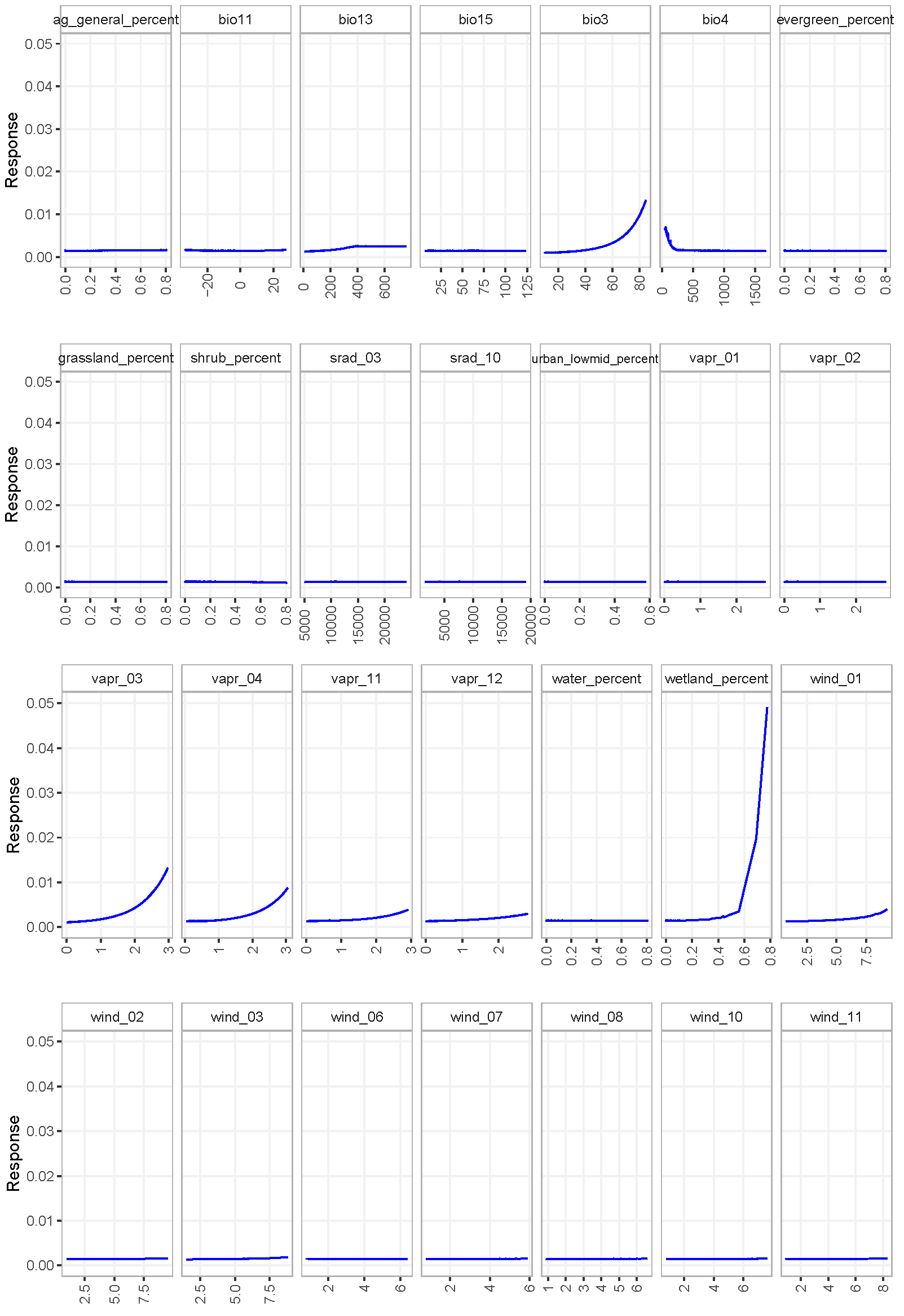

Supplement: Supplementary file 1 [file microorganisms-12-01898-s001.zip › FigureS2.png]

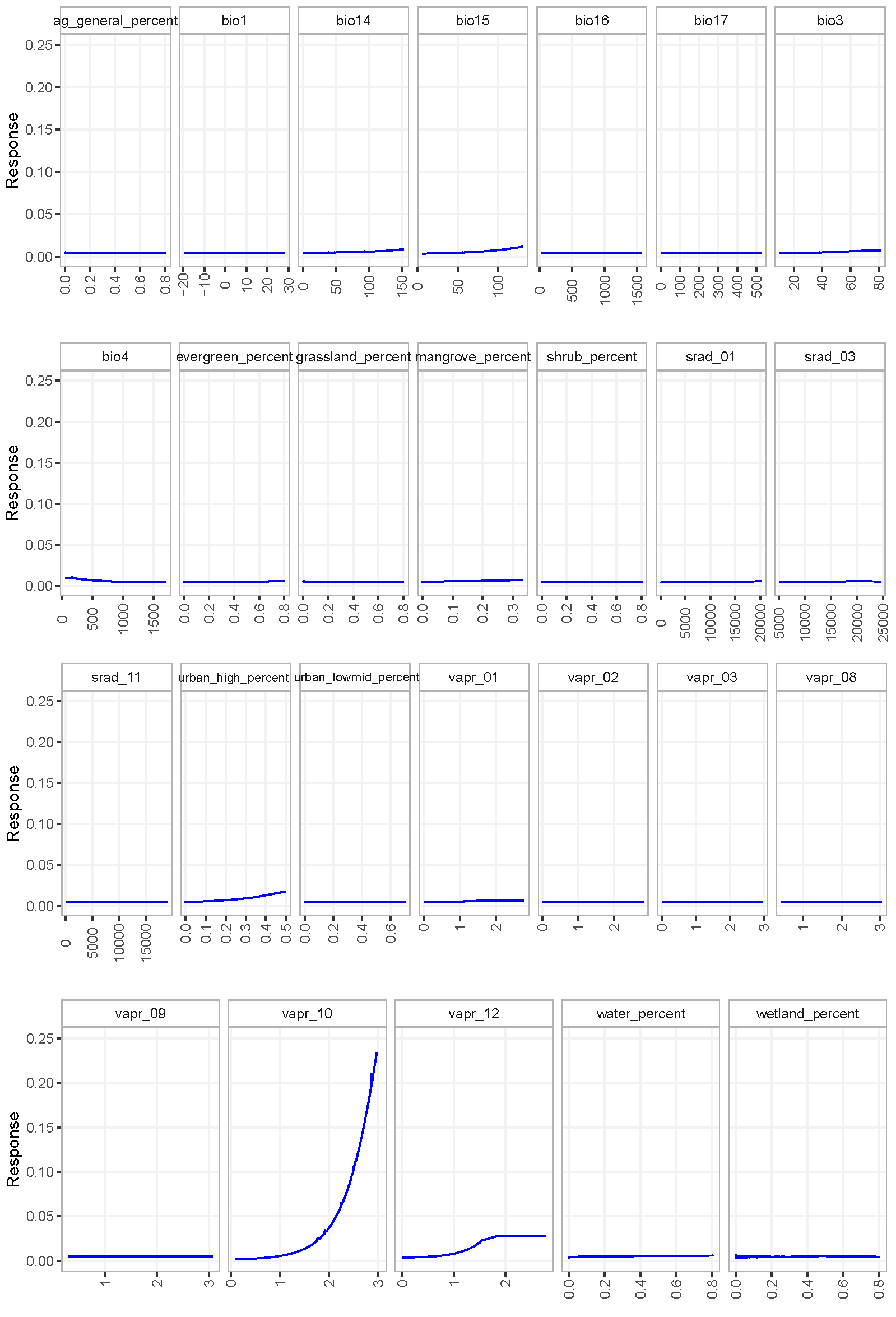

Supplement: Supplementary file 1 [file microorganisms-12-01898-s001.zip › FigureS3.png]

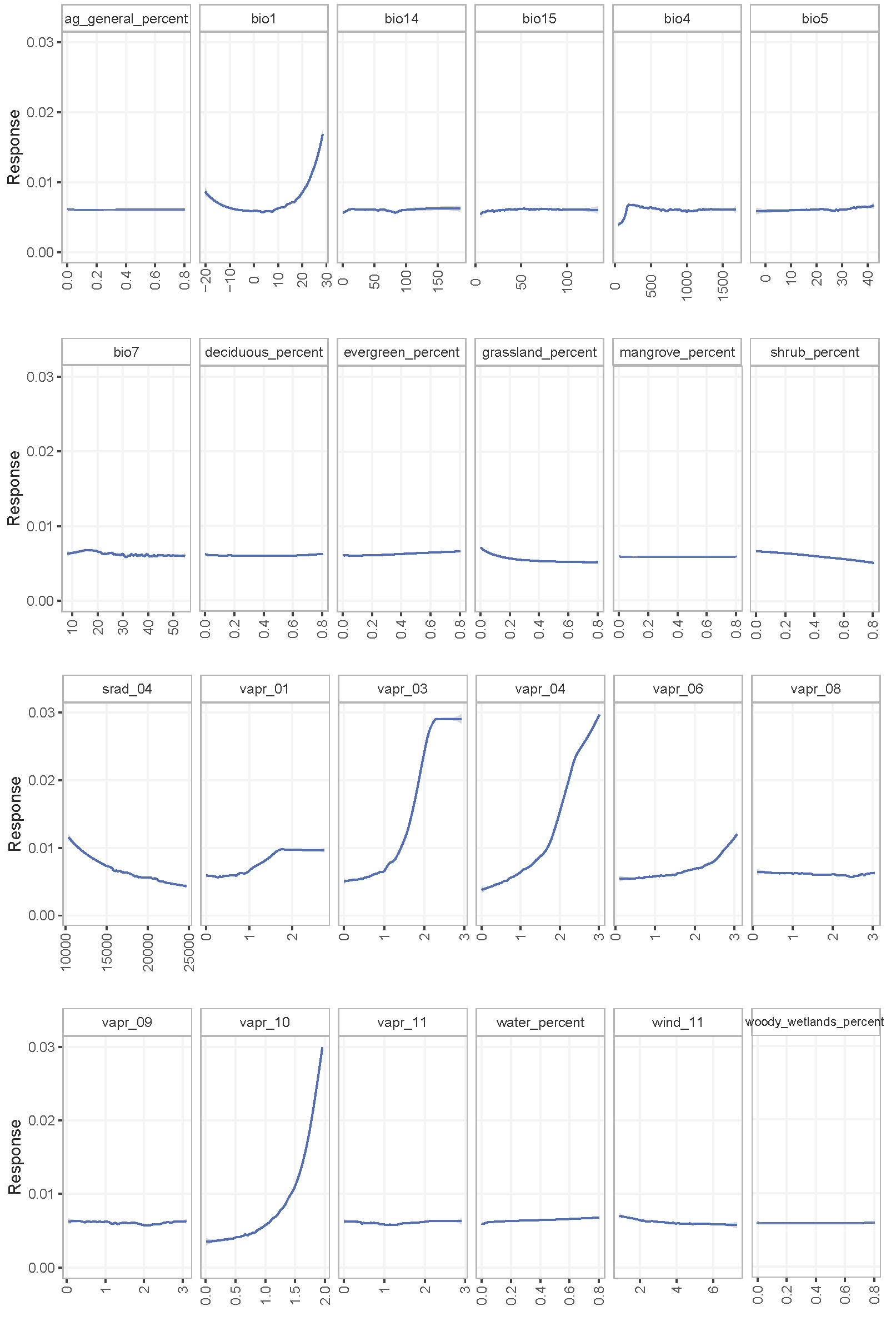

Supplement: Supplementary file 1 [file microorganisms-12-01898-s001.zip › FigureS4.png]

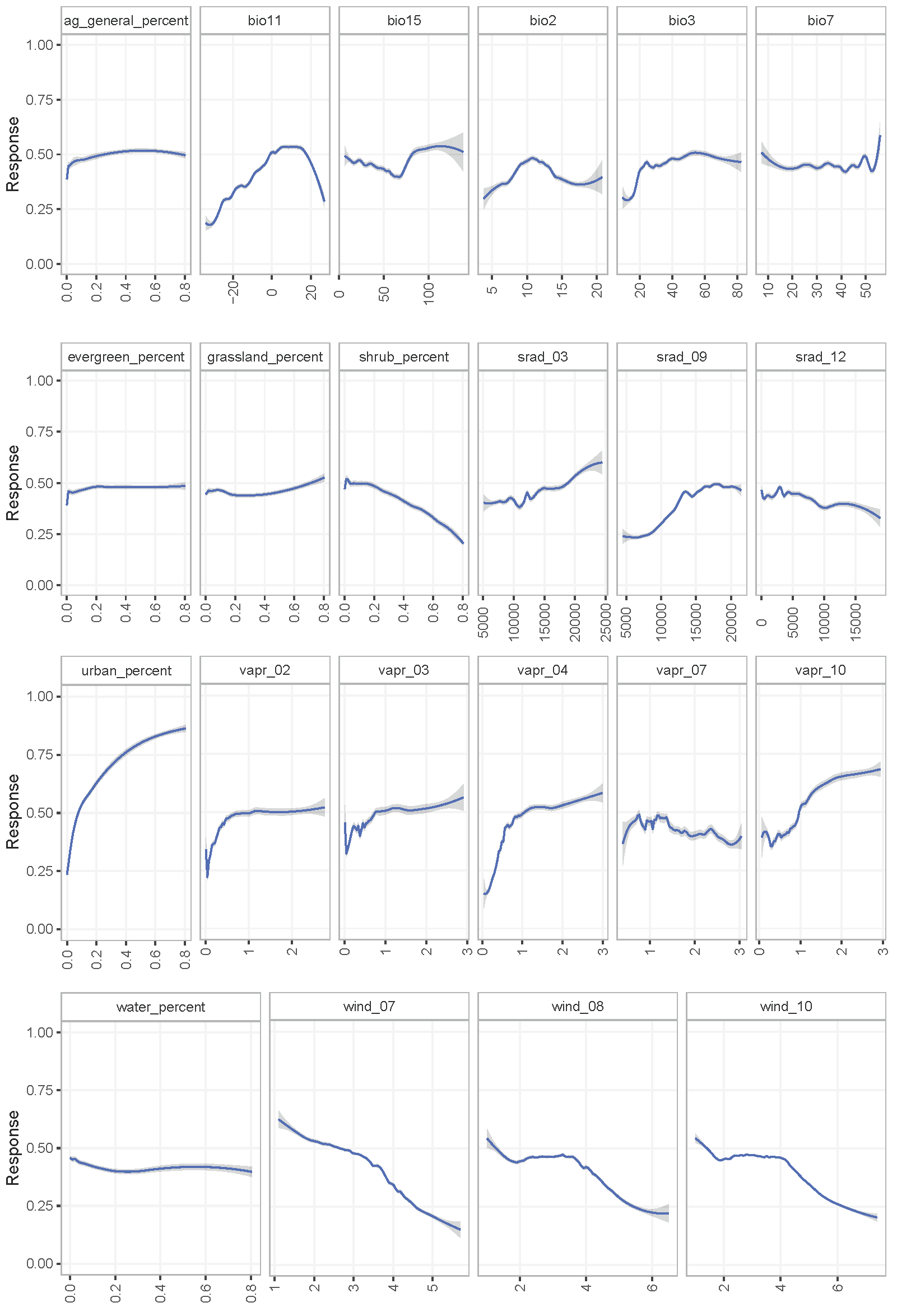

Supplement: Supplementary file 1 [file microorganisms-12-01898-s001.zip › FigureS5.png]

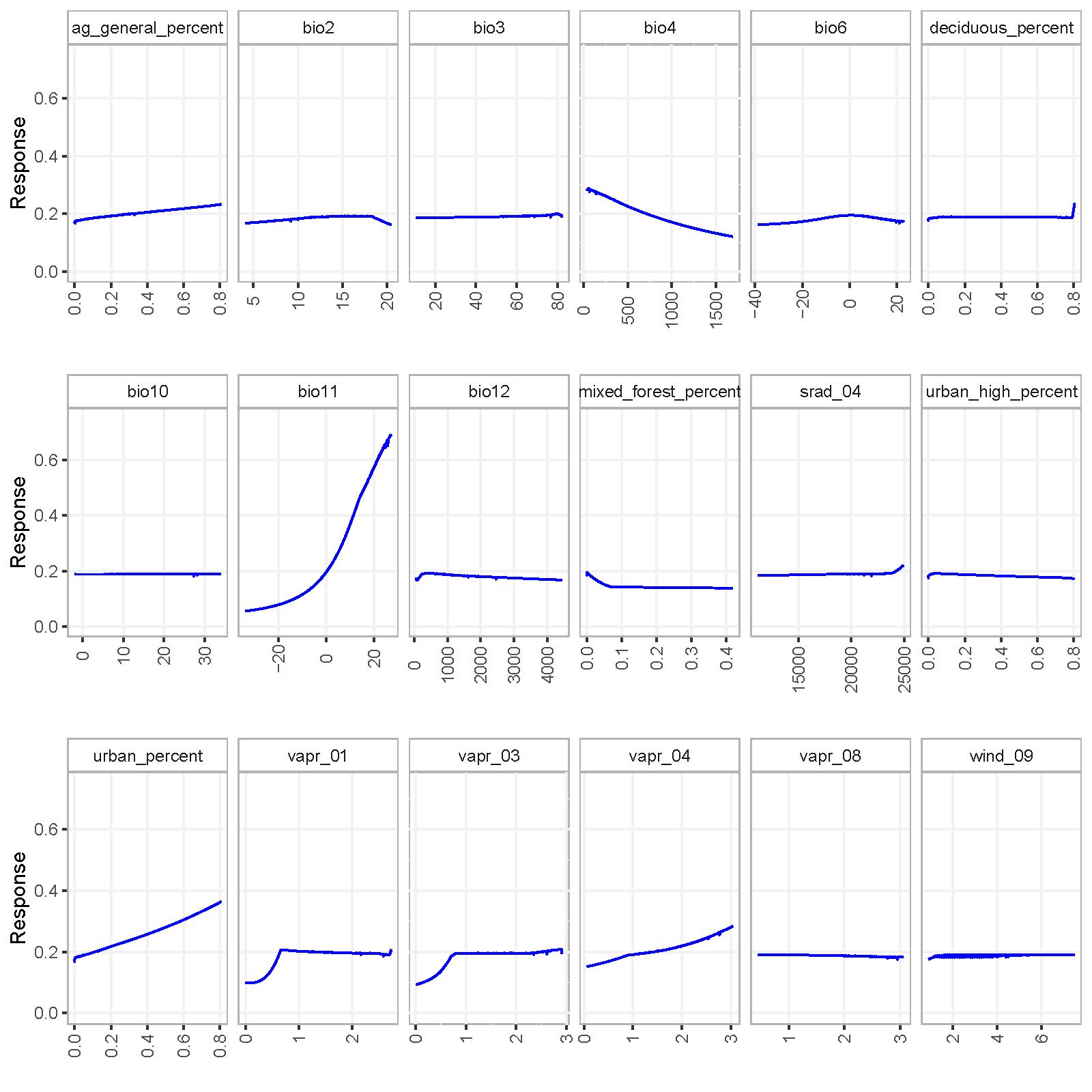

Supplement: Supplementary file 1 [file microorganisms-12-01898-s001.zip › FigureS6.png]

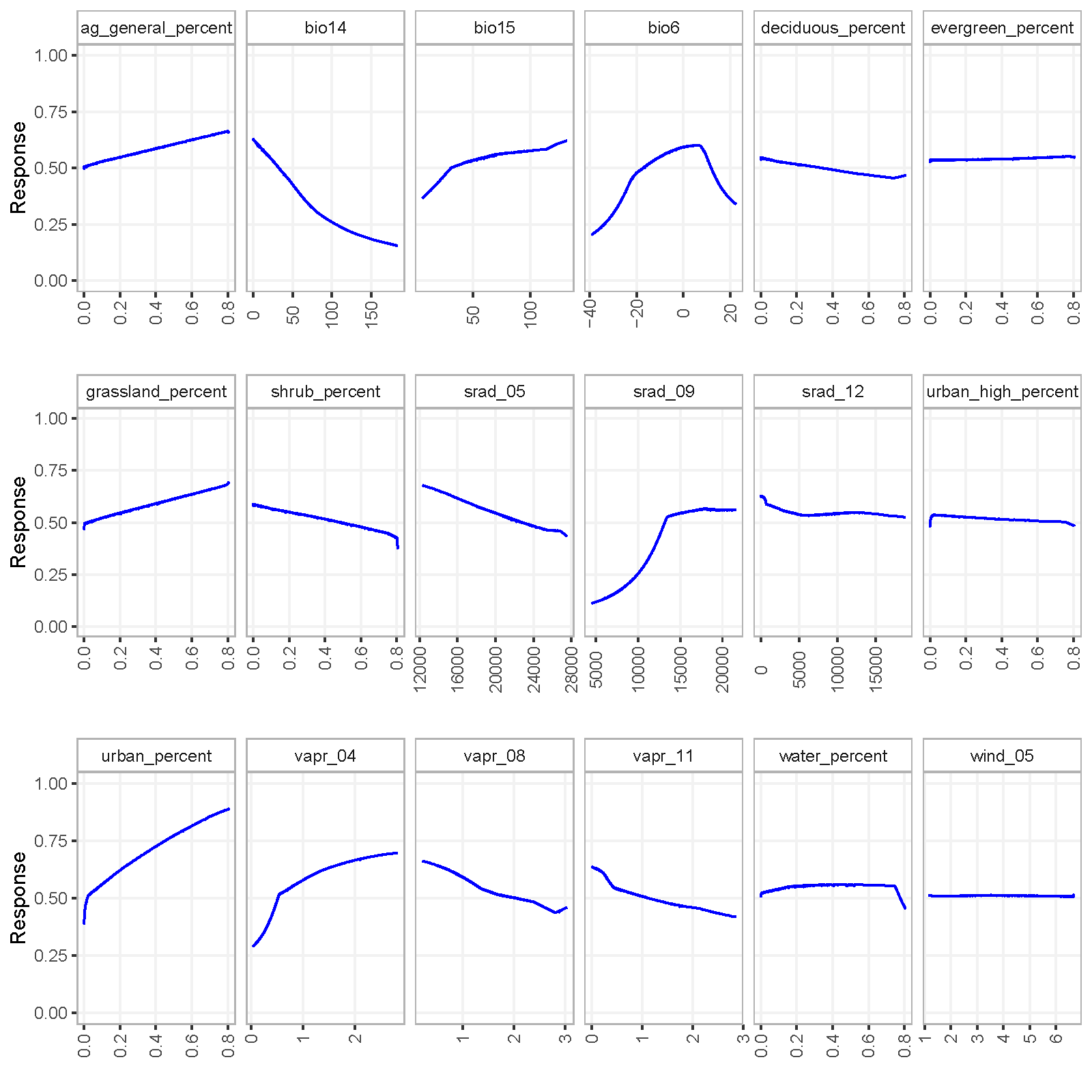

Supplement: Supplementary file 1 [file microorganisms-12-01898-s001.zip › FigureS7.png]
